# Modulatory Effects of Motor State During Paired Associative Stimulation on Motor Cortex Excitability and Motor Skill Learning

**DOI:** 10.3389/fnhum.2019.00008

**Published:** 2019-01-29

**Authors:** Jacqueline A. Palmer, Alice Halter, Whitney Gray, Steven L. Wolf, Michael R. Borich

**Affiliations:** ^1^Department of Rehabilitation Medicine, Division of Physical Therapy, Emory University, Atlanta, GA, United States; ^2^Atlanta VA Health Care System Visual and Neurocognitive Center of Excellence, Decatur, GA, United States

**Keywords:** transcranial magnetic stimulation, paired associative stimulation, motor learning, cortical excitability, plasticity, motor cortex

## Abstract

Repeated pairing of electrical stimulation of a peripheral nerve with transcranial magnetic stimulation (TMS) over the primary motor cortex (M1) representation for a target muscle can induce neuroplastic adaptations in the human brain related to motor learning. The extent to which the motor state during this form of paired associative stimulation (PAS) influences the degree and mechanisms of neuroplasticity or motor learning is unclear. Here, we investigated the effect of volitional muscle contraction during PAS on: (1) measures of general corticomotor excitability and intracortical circuit excitability; and (2) motor performance and learning. We assessed measures of corticomotor excitability using TMS and motor skill performance during a serial reaction time task (SRTT) at baseline and at 0, 30, 60 min post-PAS. Participants completed a SRTT retention test 1 week following the first two PAS sessions. Following the PAS intervention where the hand muscle maintained an active muscle contraction (PAS_ACTIVE_), there was lower short interval intracortical inhibition compared to PAS during a resting motor state (PAS_REST_) and a sham PAS condition (PAS_CONTROL_). SRTT performance improved within the session regardless of PAS condition. SRTT retention was greater following both PAS_ACTIVE_ and PAS_REST_ after 1 week compared to PAS_CONTROL_. These findings suggest that PAS may enhance motor learning retention and that motor state may be used to target different neural mechanisms of intracortical excitation and inhibition during PAS. This observation may be important to consider for the use of therapeutic noninvasive brain stimulation in neurologic patient populations.

## Introduction

Triggered by a variety of internal and external environmental stimuli, neural networks have a remarkable ability to modify their structure and function to learn new behaviors (Kandel, 2001; Cooper, 2005). Neural plasticity is an underlying mechanism for motor learning in both the neurologically intact and injured brain (Kleim and Jones, 2008). To improve motor function, various types of noninvasive brain stimulation to induce positive neural plasticity in the injured brain and as a primer for rehabilitation have been studied (Player et al., 2012; Carson and Kennedy, 2013). One type, paired associative stimulation (PAS), involves the repetitive close pairing of an electrical stimulus of a peripheral nerve with transcranial magnetic stimulation (TMS) of the contralateral primary motor cortex (M1). Through spike-timing dependent plasticity (STDP) and the induction of long-term potentiation (LTP)-like processes, changes in corticomotor excitability can be induced with this repeating pairing over time (Müller-Dahlhaus et al., 2008). The direction and magnitude of the induced neurophysiologic effects of PAS are: largely dependent on the interstimulus interval (Wolters et al., 2003); highly variable between individuals (Müller-Dahlhaus et al., 2008; López-Alonso et al., 2014); observed beyond the period of stimulation (Stefan et al., 2002; Player et al., 2012); and affected by the state of the motor system (rest vs. muscular contraction) within and homologous to the targeted limb (Kujirai et al., 2006; Kennedy and Carson, 2008; Koch et al., 2013).

While increased general corticomotor excitability is commonly observed after facilitatory PAS, as indicted by increased amplitude of the motor-evoked-potential (MEP) in response to single-pulse TMS (for review see Carson and Kennedy, 2013), the underlying mechanisms for such increases in corticomotor drive are poorly understood. Traditional PAS techniques targeting muscles at rest can affect neuronal networks with indirect cortical inputs of facilitation and inhibition (Humeau et al., 2003; Kujirai et al., 2006; Carson and Kennedy, 2013); however, previous studies have generally found no change in measures of intracortical facilitation (ICF) and short interval intracortical inhibition (SICI) that reflect activity of these neuronal networks (Stefan et al., 2002; Sale et al., 2007; Russmann et al., 2009). Interestingly, Kujirai et al. (2006) observed that when the targeted muscle maintained a submaximal contraction throughout the course of the PAS protocol, there was a greater enhancement of general corticomotor excitability. This effect was coupled with a decrease in SICI and an increase in ICF when a sub-motor threshold TMS current directed in the anterior to posterior direction was induced to target these indirect synaptic inputs (Kujirai et al., 2006). These findings suggest that; such indirect intracortical circuits contribute to the effects of PAS on corticomotor excitability; the active state of the motor system may preferentially enhance these effects (Kujirai et al., 2006); or the active state of the cortex could target different mechanisms of cortical plasticity than those at rest (Koch et al., 2013). Interestingly, Bunday et al. (2018) recently demonstrated that producing a light volitional contraction during a PAS paradigm further enhanced corticospinal excitability compared to the same PAS paradigm performed at rest in a group of individuals with incomplete spinal cord injury. Following a PAS paradigm performed at rest, our laboratory previously found that a PAS intervention could enhance corticomotor excitability in stroke survivors and that this increase in corticomotor excitability was associated with enhanced motor skill performance following PAS (Palmer et al., 2018). Importantly, compared to neurologically-intact individuals, atypical cortical network behavior has been observed in stroke survivors during active but not resting motor states in multiple instances (Murase et al., 2004; Borich et al., 2016; Palmer et al., 2016, 2017). Additionally, intracortical neural activity indexed by SICI and ICF is associated with volitional movement and motor learning (Hall et al., 2011; Coxon et al., 2014; Morin-Moncet et al., 2018). These observations support the need for a better understanding of the potential effect of the state of the motor system on PAS-induced modulation of cortical excitability. Improved understanding of the effect of motor state on mechanisms of neuromodulation could have important implications for clinical translation of effective PAS protocols to neurologic patient populations. Importantly, the relationship between these differences in cortical mechanisms in active vs. resting motor states and changes in motor performance and learning have not been well characterized.

The fundamental goal of therapeutic noninvasive brain stimulation techniques, such as PAS, is to induce corticomotor plasticity that will improve the performance of motor tasks and increase the potential for motor learning and restoration of function. Yet, there is a paucity of research investigating the effect of PAS on motor performance and learning. Similar to mechanisms that likely mediate PAS-induced neuroplasticity (Stefan et al., 2000; Carson and Kennedy, 2013; Vallence et al., 2013), there is evidence that motor learning is associated with LTP and LTD-like processes (Rioult-Pedotti et al., 1998, 2000; Sanes and Donoghue, 2000; Ziemann et al., 2004). Interestingly, baseline performance on a motor learning task was not associated with PAS-induced increases in corticomotor excitability in young healthy individuals (Frantseva et al., 2008; Player et al., 2012; Vallence et al., 2013). Despite the lack of association between baseline motor learning performance and PAS-induced corticomotor excitability, facilitatory PAS, performed prior to a bout of motor training, could increase corticomotor excitability *via* LTP-like mechanisms. This facilitation might influence subsequent motor performance and retention through “priming” of the neuromotor pathways by which motor learning occurs. However, to our knowledge, no studies have investigated the effect of motor state during PAS on motor performance or learning.

Given the limited understanding of how the state of the motor system during PAS affects PAS-induced LTP-like neuroplasticity and motor learning, the primary purposes of this study were to: (1) investigate the effect of motor state on PAS-induced changes in corticomotor and intracortical excitability; and (2) characterize the effect of an active vs. resting motor state during a PAS intervention on motor skill acquisition and retention.

## Materials and Methods

Fifteen neurologically intact, right-handed adults (age: 24.5 ± 0.82 years, nine female) were recruited for this study. Participants were included if they were between the ages of 18–35 years, had no history of neurologic pathology, and no contraindications to TMS testing (Rossini et al., 2015). All participants gave written informed consent in accordance with the Declaration of Helsinki. All study procedures were approved by the Emory University Institutional Review Board. All participants completed three study visits separated by 1 week. During each visit, one bout of PAS (PAS_ACTIVE_, PAS_REST_, PAS_CONTROL_) was performed with the order of PAS condition randomized. The assessments detailed below were performed at time points before and after PAS. Motor skill retention was assessed at the PRE testing time point of the subsequent condition 1 week later.

### Assessment of Median Nerve Somatosensory Evoked Potentials (SEPs)

A bar electrode was placed over the left median nerve with the cathode placed proximally and the distal end of the electrode aligned with the wrist crease. Participant report of a tingling sensation in the hand area of innervation was used to confirm the electrode location. Next, electric stimuli (200 μs square wave, monophasic pulse) were delivered using a constant current stimulator (DS7A, Digitimer Ltd.) at increasing intensities until an M-wave of 1 mV in peak-to-peak amplitude was evoked in the left abductor pollicis brevis (APB) muscle. Two hundred stimuli were delivered at 3 Hz while the left hand and APB were resting. Resting state of the APB was confirmed by online monitoring of continuous EMG signals. Electroencephalography (EEG) data were continuously recorded (sampling frequency: 5,000 Hz, impedance: <5 kΩ, frequency range: 0–1,000 Hz, 0.5 μV/bit resolution) using a 32-channel TMS-compatible electrode cap (Easy Cap) and amplifier (BrainAmp DC, Brain Products Ltd.). We calculated the latency of the mean peak of the N20 component of the SEP using custom Matlab functions and EEGLAB (Delorme and Makeig, 2004) in the electrode overlying the primary somatosensory cortex (S1) in the right hemisphere (CP4). The individual N20 latency was used to determine the interstimulus interval between the peripheral nerve stimulus and TMS during PAS delivery and the assessment of short afferent inhibition (SAI) for each participant individually.

### Assessment of Corticomotor Excitability

**Figure 1:**
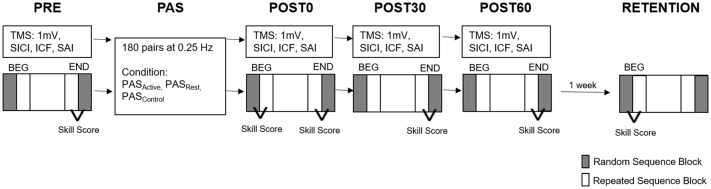
Experimental paired associative stimulation (PAS) paradigm. Each PAS protocol consisted of 180 pulse pairs using an interstimulus interval of N20 + 5 ms at 0.25 Hz delivered over the median nerve and primary motor cortex (M1). Measures of corticomotor excitability (1 mV, SICI, ICF, SAI) and motor behavior were assessed at baseline (PRE), immediately following PAS (POST 0) and at 30 and 60 min following PAS (POST30 an POST60). Motor behavior was also reassessed at a RETENTION time point approximately 1 week following POST60 in a subset of individuals. Skill Score was calculated as the difference between the random sequence block (gray) and repeated sequence block (white) at the BEG or END of each testing time point (SICI, short interval cortical inhibition; ICF, intracortical facilitation; SAI, short afferent inhibition; BEG, Beginning block; END, End block).

Following PAS delivery, corticomotor excitability was assessed at PRE, POST0, POST30, and POST60 min time points (Figure 1). Participants were seated comfortably in an upright position while monophasic magnetic pulses with a 100 μs approximate rise time and a 1.0 ms total pulse duration were delivered through a 70 mm hand-held figure-of-eight coil connected to two Magstim 200^2^ stimulators through a Bistim module (MagStim Ltd., Wales, UK). The coil was oriented perpendicular to the central sulcus to induce a posterior-anterior current in the M1 of the right (non-dominant) hemisphere (Devanne et al., 1997). Using surface electromyography (EMG), cortical excitability was evaluated by measuring the peak-to-peak amplitude of the MEP response following the stimulus artifact. EMG activity was recorded using surface electrodes (9 mm diameter, 4–5 mm inter-electrode distance) that were carefully positioned and affixed to the skin overlying the left and right APB muscle bellies, with the electrodes aligned parallel to the muscle fiber orientation. EMG data were sampled using a 16-channel EMG system (BrainAmp ExG amplifier, Brain Products GmbH) at a rate of 5,000 Hz, and band-pass filtered at 10–1,000 Hz. Using each participant’s high-resolution T_1_ anatomical MRI image (TR = 7.4 ms, TE = 3.7 ms, flip angle *θ* = 6°, FOV = 256 mm, 160 slices, 1 mm thickness), stereotactic neuronavigation software (BrainSight^®^, Rogue Research Inc., Montreal, QC, Canada) was used during TMS to ensure coil placement and orientation remained consistent for all TMS assessment measures and the PAS intervention. The optimal site for TMS was determined for each participant using standard procedures (Devanne et al., 1997). First, the stimulation intensity defined as the percent of maximum stimulator output (MSO) needed to produce *a* > 50 μV peak-to-peak amplitude MEP response in at least three out of five trials was determined as the resting motor threshold (RMT) for each participant. The TMS intensity (%MSO) needed to produce a ~1 mV MEP response in three out of five trials was also identified (Nitsche et al., 2007; Castel-Lacanal et al., 2009; Player et al., 2012). At each assessment, TMS was delivered over the right M1 APB hotspot at a rate jittered from 0.25 to 0.1 Hz while 20 MEPs were obtained for each TMS condition. Given attention levels can affect the corticomotor response (Stefan et al., 2004), a visual stimulus was presented on the computer screen positioned directly in front of the participant at randomly timed intervals. The participant counted the number of visual stimuli and reported the number after each assessment. The following TMS assessments were performed.

#### Single-pulse 1 mV MEP Response

TMS intensity was set to the ~1 mV threshold identified at the beginning of the session and held constant for all assessments within the session.

#### Short Afferent Inhibition (SAI)

When an afferent stimulus applied to a peripheral nerve precedes TMS to the contralateral M1 by ~20 ms in the assessment of hand muscles, there is typically a diminution of the MEP amplitude. These paired-pulse stimulation parameters can be used to evaluate the influence of afferent input to S1 on corticomotor output through an assessment paradigm termed SAI (Tokimura et al., 2000). Single electrical stimuli applied over the left median nerve preceded single TMS pulses over the right M1 at an interstimulus interval of the individual N20 latency plus 3 ms between the median nerve (intensity: ~1 mV M wave). An M1 stimulation intensity of ~1 mV MEP was used (Udupa et al., 2014).

#### Short Interval Intracortical Inhibition (SICI) and Intracortical Facilitation (ICF)

When a subthreshold TMS pulse precedes a suprathreshold TMS pulse using a paired-pulse paradigm, the observed MEP response is either suppressed or facilitated, depending on the interstimulus interval duration between the paired pulses (Kujirai et al., 1993; Di Lazzaro et al., 2006; Coxon et al., 2014). The terms SICI and ICF refer to the MEP response inhibition and facilitation, respectively, and are used to assess inhibitory and facilitatory intracortical components of general corticomotor excitability (Kujirai et al., 1993; Di Lazzaro et al., 2006; Coxon et al., 2014). In the present study, the interstimulus intervals between the conditioning and test TMS pulses were set to 2 ms (SICI) and 12 ms (ICF; Di Lazzaro et al., 2006). The conditioning stimulus was set at an intensity of 80% of RMT while the test stimulus was set at the same ~1 mV MEP intensity (Ziemann et al., 1996).

### Peripheral Nerve Stimulation

All MEP data were normalized to the maximal response to peripheral nerve stimulation (M-max) in the APB, enabling comparison of the normalized MEP amplitudes across PAS testing conditions. The left median nerve was stimulated with electrical pulses of gradually increasing current intensities until a plateau in the M response was achieved. Twenty pulses were delivered at the intensity needed to produce M-max at each assessment.

### Paired Associative Stimulation (PAS) Paradigm

During each PAS session, participants were seated comfortably with arms resting in a pronated position on a pillow that rested on a table set just below the level of the chest. The left median nerve was stimulated at the same intensity used for SAI assessment procedures and was held constant for the duration of the PAS session. TMS was delivered over the right M1 motor hotspot for the left APB and the intensity was set to the ~1 mV threshold for the left APB muscle, as described above. The stimulation intensity of the peripheral nerve and TMS was held constant between each PAS condition. This procedure allowed for normalization of the dosage of neurostimulation delivered during PAS intervention between each condition. A single electrical pulse applied over the left median nerve was combined with single-pulse TMS (PAS_REST_ and PAS_ACTIVE_) or sham TMS (PAS_CONTROL_) over the right M1. The interstimulus interval between the median nerve and M1 stimulation was the individual N20 latency (group mean 19.5 ± 1.1 ms) plus 5 ms to account for conduction time between S1-M1 cortico-cortical connections (Goldring et al., 1970; Conde et al., 2013; PAS_N20+5_). Each PAS session consisted of 180 pairs of stimuli (median nerve and TMS) delivered at 0.25 Hz. During the PAS_REST_ session, participants kept the APB muscle relaxed bilaterally (verified by monitoring of continuous EMG activity). Throughout the PAS_ACTIVE_ and PAS_CONTROL_ conditions, participants maintained a light contraction of APB at 15% of their maximum EMG activity. Participants were provided continuous visual feedback of APB EMG activity on a computer display to ensure that they maintained a consistent level of muscle activation. Additionally, participants were instructed to count the number of visual light stimuli presented directly over the left APB in all conditions to account for attentional effects on PAS response (Stefan et al., 2004). The visual light stimulus was presented at random intervals between 5 and 20 times total during the PAS intervention. The visual stimulus was not presented during PAS delivery but provided between PAS pairs to avoid possible interactions between visual information processing and PAS-evoked cortical activity.

### Assessment of Motor Behavior

A unimanual version of the serial reaction time test (SRTT) was used to characterize the effects of PAS on motor skill performance (Cohen et al., 2005). Participants were seated in front of a standard keyboard. A computer screen at eye-level displayed a row of white rectangular targets corresponding to each of four sequential keys (V, B, N, M) on the keyboard. The participants were told the task evaluated reaction time and were instructed to press the correct key with the corresponding digit of the non-dominant hand (e.g., press “V” with the 5th digit when the leftmost target was illuminated on the screen) as quickly and accurately as possible. After a correct response, a new stimulus target was illuminated following a delay of 400 ms. For each assessment time point, participants completed 17 blocks (280 key presses total) of the SRTT. During the first and last block, a 50-element stimulus sequence was displayed in random, non-repeating order. Each middle block (blocks 2–16) repeated an identical 12-element sequence (B-N-V-M-N-B-M-V-N-M-B-V). The duration of each SRTT assessment was dependent on the speed at which the participant completed the key presses and typically lasted approximately 3 min. The SRTT was performed with the left hand at baseline (PRE), POST0, POST30, and POST60 testing time points. Additionally, the SRTT assessment performed at the PRE time point during visits 2 and 3 was used as the retention assessment for visits 1 and 2 respectively. A separate retention test was not performed after visit 3.

### Primary Outcome Measures

#### Corticomotor Excitability

The peak-to-peak MEP amplitudes were averaged at each time point and normalized to the mean M-max collected at the corresponding time point. Mean amplitudes of SAI, SICI and ICF were expressed as a ratio of conditioned over unconditioned (1 mV) normalized MEP responses (MEP ratio). The mean MEP ratio was expressed as a percentage of the baseline value for each post-PAS time point.(Nitsche et al., 2007; Player et al., 2012) Percent change relative to the baseline was calculated for each MEP variable as [(POST-PRE)/PRE] * 100 (Nitsche et al., 2007; Player et al., 2012).

#### Motor Skill Performance

The response time (RT; reaction time + movement time) was defined as the time between the visual stimulus presentation and the correct key press. The mean RT during the second random sequence block (Rand_END_) was used to evaluate general motor skill performance at each assessment. A sequence-specific skill score was calculated as the difference between the average RT during the last random block and the average RT of the preceding four repeated sequence blocks of the left hand (Rand_END_ − Rep_END4_) for PRE, POST0, POST30, and POST60 time points (Figure 1). We used the RT during the 50 random trials at the beginning of the POST0 assessment (Rand_BEG_) to evaluate the immediate effects of PAS on general motor skill performance before additional task practice. To investigate the immediate effects of PAS on sequence-specific motor performance, we also calculated the skill score at the beginning of the POST0 SRTT as the difference between mean RTs for Rand_BEG_ and Rep_BEG4_.

#### Motor Skill Retention

The influence of PAS on motor skill retention was evaluated. We calculated an additional RETENTION time point skill score as the difference between the average RT of the first random block and the average RT of the next four repeated sequence blocks (Rand_BEG_ − Rep_BEG4_) for the PRE assessment performed on visit 2 and visit 3 as a basis for evaluating skill retention from visit 1 and visit 2, respectively. The difference between RETENTION skill score and POST60 skill score from the end of the preceding session was used to quantify level of sequence-specific skill retention. Performance on the first random block at retention (Rand_BEG_) was compared to performance on the last random block at POST60 (Rand_END_) to evaluate general motor performance retention (Figure 1).

### Statistical Analyses

Each primary outcome measure was tested for normality and homogeneity of variance using Kolmogorov-Smirnov and Levene’s tests, respectively. Parametric testing procedures were performed if data met assumptions of normality and homogeneity of variance as indicated by a non-significant (*p* > 0.05) test result. Separate repeated measures analysis of variance (RM-ANOVA) tests with within-subject factors of time (POST0, POST30, POST60) and condition (PAS_ACTIVE_, PAS_REST_, PAS_CONTROL_) were used to test the effect of PAS and muscle activation on corticomotor excitability for all MEP measures (1 mV, SAI, SICI, and ICF).

The following tests were performed to assess motor skill:

1. *Immediate effect of PAS condition on motor performance*: to test the immediate within-session effect of PAS condition on sequence-specific motor performance and general sequence motor performance, separate (time × condition) RM-ANOVAs were performed for the skill score and the RT during random sequence blocks, respectively.
2. *Effect of PAS condition on motor performance over time within session*: to test the effect of PAS on general and sequence motor performance over time within the session, RT during the random block and skill score at the end block over all within-session time points during each condition using a two-way RM-ANOVA were compared.
3. *Effect of PAS condition on motor retention*: to test the effect of PAS condition on retention of motor performance, the random RT and skill score of the POST60 end block vs. the RETENTION beginning blocks of follow-up visits were compared using a two-way ANOVA (Figure 1). For all significant interactions and main effects, *post hoc* pairwise testing was performed. All analyses were undertaken using SPSS version 24 with an uncorrected α level set to 0.05.

## Results

Complete data sets for corticomotor excitability were obtained for all participants in both PAS_ACTIVE_ and PAS_REST_ conditions, except for one participant who did not complete TMS testing at the POST60 time point following PAS_ACTIVE_ due to time constraints and one participant who did not complete peripheral nerve stimulation for any time point during the PAS_REST_ session. Two participants did not complete the PAS_CONTROL_ condition and were lost to follow-up. These data points were not available for the corresponding analyses resulting in 13 complete data sets for analyses. Motor behavioral testing was completed for all participants at all within-session time points for both PAS_ACTIVE_ and PAS_REST_ conditions and for all but two participants who did not complete the PAS_CONTROL_ condition. All corticomotor excitability and motor skill data met assumptions of normality and homogeneity of variance.

### Corticomotor Excitability

**Figure 2:**
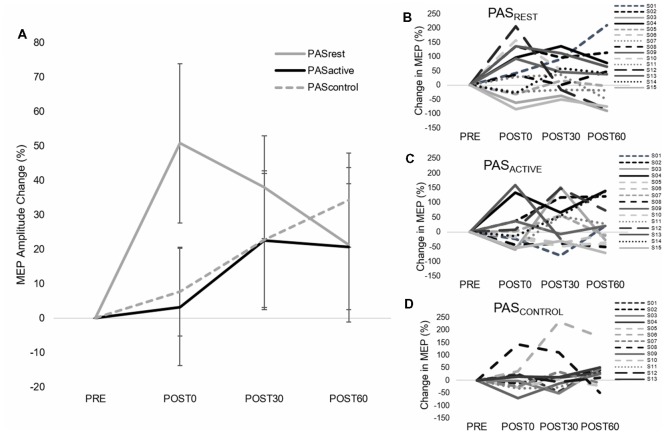
Group (left) and individual (right) motor-evoked-potential (MEP) amplitude (mean ± SE) during single-pulse 1 mV transcranial magnetic stimulation (TMS) assessment. **(A)** Average MEP amplitude change for each PAS condition. **(B–D)** Individual MEP amplitude change for each PAS condition. There was no condition-by-time interaction (*F*_(4,14)_ = 1.12, *p* = 0.36) or main effects of PAS condition (*F*_(2,14)_ = 1.17, *p* = 0.33) or time (*F*_(2,14)_ = 1.59, *p* = 0.21).

**Figure 3:**
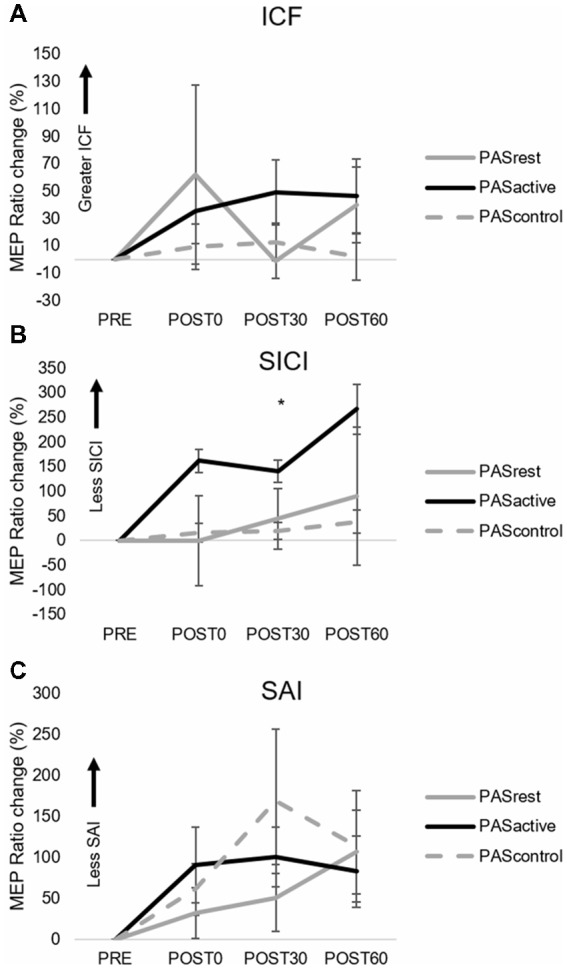
Group normalized MEP amplitude (mean ± SE) during the PAS_ACTIVE_, PAS_REST_, and PAS_CONTROL_ conditions for **(A)** ICF, **(B)** SICI, and **(C)** SAI TMS assessments. MEP ratio measures for SICI **(B)** demonstrated a main effect of condition (*p = 0.048), where PAS_ACTIVE_ showed greater increase in MEP amplitude across post-testing time points compared to PAS_REST_ and PAS_CONTROL_ (SAI, short afferent inhibition; SICI, short interval intracortical inhibition; ICF, intracortical facilitation).

When testing the effects of PAS condition on 1 mV MEP amplitude change over time, we did not observe a condition-by-time interaction (*F*_(4,14)_ = 1.12, *p* = 0.36) or main effects of PAS condition (*F*_(2,14)_ = 1.17, *p* = 0.33) or time (*F*_(2,14)_ = 1.59, *p* = 0.21; Figure 2). Similarly, there was no interaction effect (*F*_(4,14)_ = 1.39, *p* = 0.24) or main effects of time (*F*_(2,14)_ = 0.01, *p* = 0.99) or condition (*F*_(2,14)_ = 0.93, *p* = 0.41) on relative change in ICF (Figure 3A). For SICI measures, analyses revealed a main effect of condition (*F*_(2,14)_ = 3.34, *p* = 0.048), with lower SICI (ratios closer to 1) observed across POST assessment time points following PAS_ACTIVE_ compared to PAS_REST_ and PAS_CONTROL_ (Figure 3B). We did not observe a significant interaction (*F*_(4,14)_ = 0.54, *p* = 0.71) or main effect of time (*F*_(2,14)_ = 1.6 *p* = 0.24). For change in SAI, there was no interaction (*F*_(4,14)_ = 1.76, *p* = 0.16) nor a main effect of condition (*F*_(2,14)_ = 0.24, *p* = 0.79) or time (*F*_(2,14)_ = 0.19, *p* = 0.83) on change in SAI (Figure 3C).

### Motor Skill Performance

**Figure 4:**
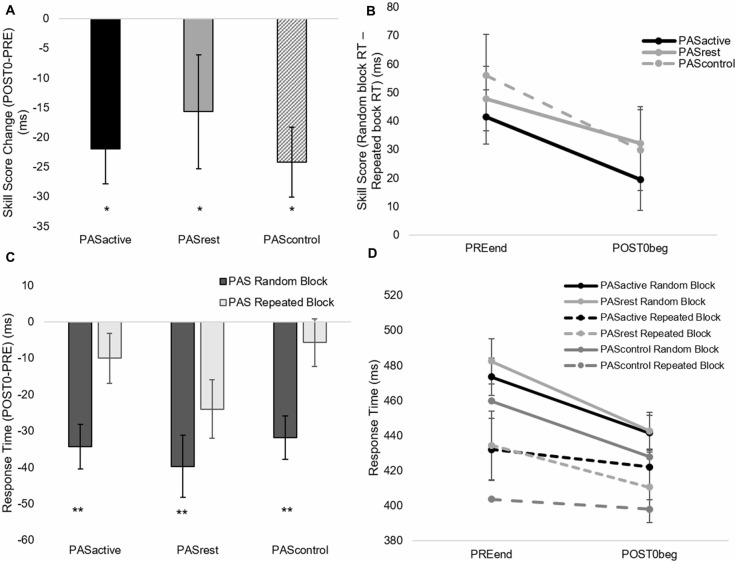
Immediate effect of PAS condition on motor performance. Skill score **(A,B)** and random sequence response time (RT; **C,D**) calculated from serial reaction time task (SRTT) performance (mean ± SE) during PAS_ACTIVE_, PAS_REST_ and PAS_CONTROL_ conditions at PRE_END_ and POST0_BEG_ time points. Skill score decreased (*p < 0.05) while random sequence RT improved (reduced RT; **p < 0.001) immediately following all PAS conditions.

**Figure 5:**
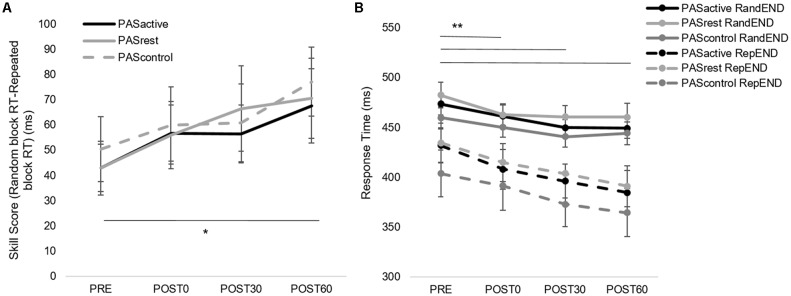
**(A)** Skill score and **(B)** random block RT during SRTT assessment (mean ± SE) for PAS_ACTIVE_, PAS_REST_,and PAS_CONTROL_ conditions for each within-session time points. Skill score improved over time (*p = 0.03) and RT decreased (**p < 0.001) in both conditions.

**Figure 6:**
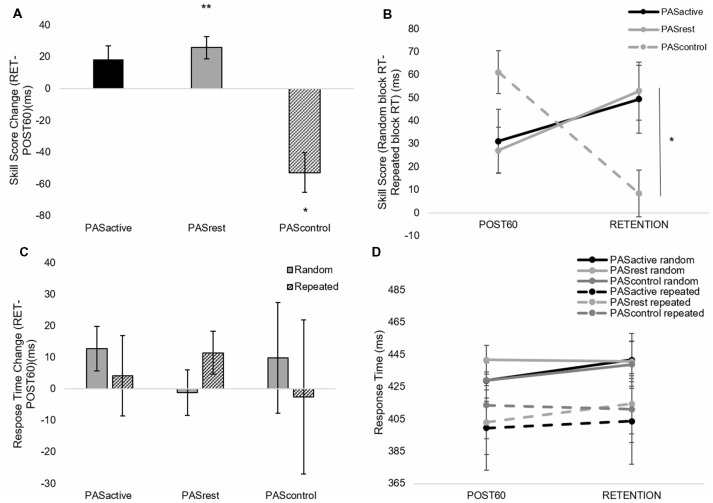
Skill score **(A,B)** and random block RT **(C,D)** calculated from SRTT performance (mean ± SE) during PAS_ACTIVE_, PAS_REST_,and PAS_CONTROL_ conditions at POST60 and RETENTION time points. There was a condition-by-time interaction for skill score (*F*_(2,50)_ = 4.31, *p* = 0.02). At retention, skill score following PAS_REST_ was greater than that following PAS_CONTROL_ (*p < 0.05) with no significant change following PAS_ACTIVE_ (*p* = 0.10). Skill score increased following PAS_REST_ (**p < 0.01) and decreased following PAS_CONTROL_ (*p < 0.05). **(C,D)** PAS condition did not significantly affect retention of general motor performance on the SRTT.

1. When testing for the immediate effect of PAS condition on motor skill performance, there was no interaction effect (skill score, *F*_(2,14)_ = 0.344, *p* = 0.71; random RT, *F*_(2,14)_ = 0.47, *p* = 0.63) or effect of PAS condition (skill score, *F*_(2,14)_ = 0.47, *p* = 0.63; random RT, *F*_(2,14)_ = 0.83, *p* = 0.45). There was a main effect of time for both RT during the random sequence (*F*_(1,14)_ = 37.13, *p* < 0.001) and skill score (*F*_(1,14)_ = 21.3, *p* < 0.01), indicating that both general motor performance and sequence-specific performance showed significant change immediately after PAS in all conditions (Figures 4A,B). Following PAS, general motor performance improved to a greater degree than sequence-specific motor performance (Figures 4C,D), contributing to the decrease in skill score observed in all conditions immediately following PAS.
2. When testing the interaction between PAS condition and motor performance over time within the session, there was no interaction effect (skill score, *F*_(6,14)_ = 0.20, *p* = 0.98; random RT, *F*_(3,14)_ = 0.39, *p* = 0.76) or effect of condition (skill score, *F*_(2,14)_ = 0.06, *p* = 0.94; random RT, *F*_(3,14)_ = 1.24, *p* = 0.29). However, there was a main effect of time for both random RT (*F*_(3,14)_ = 9.80, *p* < 0.001) and skill score (*F*_(3,14)_ = 5.1, *p* < 0.01). Results showed improvements in both general and sequence-specific SRTT performance, regardless of PAS condition (Figures 5A,B). For skill score, *post hoc* comparisons revealed greater skill score at the POST60 time point (*p* = 0.03) and a trend towards greater skill score at POST30 (*p* = 0.07) time points compared to PRE. For random sequence, *post hoc* testing showed RT decreased at all POST testing time points compared to PRE (*p* < 0.001).
3. When testing the effect of PAS condition on retention of sequence-specific performance, there was a significant condition-by-time interaction for skill score (*F*_(2,50)_ = 4.31, *p* = 0.02; Figures 6A,B). At POST60, we did not observe a significant difference in skill score between any of the three PAS conditions (PAS_ACTIVE_ vs. PAS_CONTROL_, *p* = 0.18; PAS_REST_ vs. PAS_CONTROL_, *p* = 0.05; PAS_ACTIVE_ vs. PAS_REST_, *p* = 0.83). At retention, skill score following PAS_REST_ was greater than that following PAS_CONTROL_ (*p* = 0.04) and there was a non-significant trend for differences in skill score at retention between PAS_ACTIVE_ vs. PAS_CONTROL_ (*p* = 0.09). There was no significant difference between PAS_REST_ and PAS_ACTIVE_ (*p* = 0.87). Skill score was increased at retention compared to POST60 following PAS_REST_ (*p* < 0.01) but not PAS_ACTIVE_ (*p* = 0.10). In contrast, skill score showed a significant reduction at retention compared to POST60 following PAS_CONTROL_ (*p* = 0.02). For random sequence RT, there was no interaction (*F*_(2,50)_ = 0.18, *p* = 0.83) or main effect of condition (*F*_(2,50)_ = 0.19, *p* = 0.83) or of time (*F*_(1,50)_ = 0.44, *p* = 0.51; Figures 6C,D).

## Discussion

The primary findings of this study reveal that the state of the motor system during a facilitatory PAS protocol may influence intracortical excitability. Further, results suggest that facilitatory PAS may enhance motor learning retention in able-bodied individuals but does not appear to be influenced by an active vs. resting motor state during PAS. Maintaining an active muscle contraction during PAS did not result in a change in general corticomotor excitability, but there was a significant reduction in SICI following PAS_ACTIVE_ that was not observed following PAS_REST_ or PAS_CONTROL_. Both PAS_ACTIVE_ and PAS_REST_ showed greater but comparable sequence-specific motor skill learning retention compared to PAS_CONTROL._ These findings suggest that manipulating motor state during PAS has the potential to target intracortical circuits in the M1 but without exerting a measureable effect on motor behavior.

### Effect of Muscle Contraction on Intracortical Inhibition and Facilitation Following PAS

In the current study, maintaining an active motor state during the PAS protocol had a differential effect on intracortical inhibitory mechanisms, as we observed lower SICI following the PAS_ACTIVE_ condition. We did not observe this effect on SICI following PAS_CONTROL_ or PAS_REST_,which is consistent with results reported by others following a facilitatory PAS protocol with the muscle at rest (Stefan et al., 2002; Sale et al., 2007; Elahi et al., 2012). Our results suggest that producing a voluntary contraction during a facilitatory PAS protocol could have greater effects on intracortical inhibitory circuits than on changes in the excitability of facilitatory circuits, as no changes were observed in ICF. Volitional muscle contraction might have enhanced the efficiency by which proprioceptive afferent input is transmitted to the cortex (Kunesch et al., 1995). Application of the PAS protocol during this active motor state could have potentially strengthened synaptic connectivity of the projections of proprioceptive afferents relayed through area 3a to inhibitory intracortical neurons within M1 that contribute to SICI (Porter et al., 1990; Sailer et al., 2002). The fact that the significantly lower SICI was observed following PAS_ACTIVE_ did not translate to robust enhancement of general corticomotor excitability may be consistent with the notion that PAS-induced corticomotor excitability could have been blunted by performing the SRTT prior to PAS. While preventing LTP, motor skill learning tasks performed prior to noninvasive brain stimulation protocols can enhance LTD-like mechanisms, and thus could have contributed to lower SICI following PAS_ACTIVE_ (Rioult-Pedotti et al., 2000; Ziemann et al., 2004). This LTD-like response may be specific to horizontal cortical connections strengthened by enhanced activity of proprioceptive afferents during the active motor contraction maintained under the PAS_ACTIVE_ condition. Previous studies have reported that peripheral nerve stimulation coupled with volitional contraction of muscles innervated by the stimulated nerve produced changes in corticomotor excitability to that muscle (Khaslavskaia and Sinkjaer, 2005; Yamaguchi et al., 2012) and increased the sensitivity between neural projections of the contralateral S1 and M1 (Gandolla et al., 2014). However, interactions between the peripheral nerve stimulation, volitional muscle contraction and motor cortical stimulation all likely contributed to differences in SICI following PAS_ACTIVE_ as no such modulations were observed with peripheral nerve stimulation and volitional muscle contraction alone during the PAS_CONTROL_ condition. These findings suggest differential mechanistic corticomodulatory effects induced by muscle contraction during facilitatory PAS that could be important to consider when using such protocols in neurologic patient populations where abnormal activity of intracortical neural networks may be present.

### Effect of Muscle Contraction on General Corticomotor Excitability Following PAS

Our findings suggest that an active motor state within the targeted muscle may attenuate the immediate effects of a facilitatory PAS protocol on increased general corticomotor excitability. Immediately following the PAS protocol at the POST0 time point, MEP amplitude increased 50% in the PAS_REST_ condition and less than 6% in the PAS_ACTIVE_ and PAS_CONTROL_ conditions (Figure 2A), though no conditions produced a statistically significant change from baseline MEP amplitude. These results are in contrast those of Kujirai et al. (2006), who found that producing a small contraction in the targeted muscle enhanced the immediate PAS-induced increase in general corticomotor excitability as well as intracortical facilitation measures. Differences in results may be explained by differences in study protocols, as Kujirai et al. (2006) applied TMS at a subthreshold intensity and in an anterior-posterior direction, which preferentially targets indirect synaptic outputs. Interestingly, we did not observe a significant increase in MEP amplitude following either active or resting PAS conditions. These findings are inconsistent with some previous studies that found increased MEP amplitudes for up to 30 min following a PAS_25_ protocol (Wolters et al., 2003; Kujirai et al., 2006; Player et al., 2012). High levels of inter-individual variability in PAS-induced modulation of M1 excitability, particularly in the PAS_REST_ and PAS_ACTIVE_ conditions likely contributed to insignificant group MEP changes, which has been reported previously (Müller-Dahlhaus et al., 2008). As previously mentioned, the typically observed increase in general corticomotor excitability could have been attenuated by the motor skill learning task prior to PAS and repeated exposure to the task throughout the session, which can diminish LTP-like neuroplasticity (Rioult-Pedotti et al., 2000; Ziemann et al., 2004). Both animal and human studies have shown that motor skill learning prevented LTP typically induced by facilitatory noninvasive brain stimulation protocols, even though LTD was enhanced (Rioult-Pedotti et al., 2000; Ziemann et al., 2004). Additionally, genetic factors shown to influence LTP-like cortical plasticity and motor learning could have contributed to the high inter-individual subject variability and reduced effect sizes in corticomotor response to PAS observed in the present study (Kleim et al., 2006; Di Lazzaro et al., 2015; Morin-Moncet et al., 2018). Differences in results could also be explained, in part, by the customization of the interstimulus interval used in the present study (PAS_N20 + 5_) compared to standard intervals commonly used (e.g., PAS_25_; Wolters et al., 2003; Kujirai et al., 2006; Player et al., 2012). Customization of the interstimulus interval using each individual’s N20 latency during PAS was expected to increase the likelihood of coincident timing of inputs in M1 resulting in larger LTP-like increases in corticomotor excitability. However, it is not clear that customizing the ISI using N20 latencies will increase PAS-induced modulatory effects (Hamada et al., 2012). The N20 response is primarily a reflection of cutaneous afferents of area 3b which do not have strong projections to M1 and are not engaged to a significant degree by muscle spindle afferents believed to primarily contribute to PAS-induced increases in corticomotor excitability (Carson and Kennedy, 2013). Regardless, the ISI for each participant was within the range of ISI values for PAS protocols used in previous studies that showed significant LTP-like plasticity Future studies examining the importance of optimizing the ISI during PAS and the effect of motor skill exposure on PAS-induced changes in corticomotor and intracortical excitability will further refine the interpretation of the current findings. Additionally, findings of this study and others warrant future research investigating potential neurobiological predictors of response to noninvasive brain stimulation interventions such as PAS.

### Effects of PAS on Motor Skill Performance and Learning

General motor performance on the SRTT was improved significantly directly following PAS completion for each condition; however, sequence-specific skill showed a reduction in performance immediately after each PAS condition. As anticipated, across post-PAS assessments, there was a significant improvement in SRTT performance for both random and repeated sequences. Given the repeated task exposure at each time point, motor skill performance improvement is not surprising. There was no difference in immediate motor skill performance behavior between PAS conditions; thus, in agreement with previous findings (Lopez-Alonso et al., 2018), PAS probably does not uniquely affect immediate motor performance within a session. Though these findings may contrast those of Jung and Ziemann (2009), where improvements in rapid thumb flexion movements following PAS were observed. These differences between findings may be due to differences in motor performance assessment (ballistic finger movement vs. skilled sequential motor performance). Thus, the effect of PAS on motor performance may depend to some extent on the characteristics of the task employed and how motor skill performance is evaluated. Interestingly, sequence-specific skill retention was preferentially enhanced following PAS_REST_ with a trend towards enhancement following PAS_ACTIVE_. In contrast, skill performance was significantly lower at retention following PAS_CONTROL_. These findings contrast with those of Lopez-Alonso et al. (2018) who found no differences in motor performance on an isometric pinch force tracking task 1 week following PAS performed at rest compared to a sham condition. Differences between these findings and results of a present study could also be explained by differences in the attributes between motor tasks and/or the neural substrates involved in each task. Thus, taken together, findings support the notion that PAS may differentially affect distinct neural pathways involved in learning motor skills with different task characteristics. Our current findings together with previous literature on PAS-induced effects on different aspects of motor performance and learning could offer valuable information for rehabilitation interventions targeting different aspects of motor coordination and control.

Despite no preferential enhancement of within-session motor performance, PAS may target and promote salient mechanisms underlying sequence-specific motor skill learning. Although intracortical inhibition was lower following PAS_ACTIVE_, changes in corticomotor excitability measures likely do not primarily contribute to greater motor skill retention following both PAS_ACTIVE_ and PAS_REST_, as these changes in corticomotor measures were not observed in both PAS conditions. Few studies have investigated the immediate effect of PAS on motor learning or performance (Player et al., 2012; Lopez-Alonso et al., 2018) and, to our knowledge, this study is the first to investigate the effect of motor state during PAS on delayed motor skill retention. PAS may have enhanced experience-dependent neuroplasticity resulting in improved motor retention that was not captured by TMS assessments of M1 cortical excitability. The fact that significant modulation of general corticomotor excitability was not demonstrated for both PAS_REST_ and PAS_ACTIVE_ conditions implies that other neural mechanisms either within or outside M1 may underpin enhanced motor skill retention following PAS regardless of motor state. Quantification of neurophysiologic changes following PAS using functional, structural and/or metabolic neuroimaging approaches may identify and characterize PAS-induced neuroplastic mechanisms underlying enhanced skill retention observed in the current study. Future studies should confirm these findings and further evaluate the capacity for PAS-induced neuroplasticity to enhance the effects of motor skill training on skill performance and learning in able-bodied individuals and patients with neurologic diagnoses.

### Limitations

Several limitations could influence the interpretation of the observed results. High inter-individual variability in response to PAS for each protocol was observed, reducing statistical power in a relatively small participant cohort. Evaluation of individual response profiles may provide additional information to guide future PAS interventions. Future studies could incorporate an inhibitory PAS condition to evaluate the effects of PAS on cortical excitability and motor skill learning. Though the SRTT is a valid and reliable motor task for detecting changes in sequence specific motor performance (Cohen et al., 2005), the task was not specific to the targeted muscle during PAS. However, the reduced spatial anatomical selectivity of suprathreshold TMS combined with overlap in digit representations in M1 reduces the likely importance of this limitation. The small sample size and thus the preliminary nature of the results warrants future studies to explore further the effect of PAS on motor skill retention and its relationship to changes in neurophysiologic measures. Due to the time constraints and number of measures during the session, corticomotor assessments of the M1 ipsilateral to the target muscle were not performed. Kennedy and Carson (2008) found that contraction of the muscle homologous to the targeted muscle in the contralateral limb could produce differential PAS-induced effects within the contralateral limb. Investigating potential corticomotor changes in the ipsilateral hemisphere following PAS could provide useful and important information in future studies. The present study controlled for possible changes at the neuromuscular junction in response to the intervention by normalizing all MEP data to the Mmax; however, we did not measure spinal or subcortical mechanisms. Modulation of MEP amplitude observed in the present study could be attributed to differences in interactions with local subcortical or spinal circuits rather than to cortical mechanisms. Future studies are required to advance our understanding of the mechanisms underlying the neuromodulator effects of PAS-based interventions in both health and disease.

When performed during an active motor state, PAS can transiently reduce intracortical inhibition, though this reduction does not appear to preferentially augment motor skill performance or learning in able-bodied individuals. Irrespective of the state of the motor system, facilitatory PAS increased sequence-specific motor learning suggesting PAS could offer a neuromodulatory tool to enhance response to skill training and/or motor rehabilitation. Current findings may inform the development of future PAS approaches targeting atypical corticomotor and intracortical excitability to augment therapeutic response in neurologic patient populations to improve motor function and reduce disability.

## Author Contributions

JP: data collection, data processing/analysis, statistical analysis, original manuscript drafting, manuscript editing. AH and WG: data collection, data processing, manuscript revision. SW: project conception, research design, manuscript revision. MB: project conception, research design, data collection, manuscript revision.

## Conflict of Interest Statement

The authors declare that the research was conducted in the absence of any commercial or financial relationships that could be construed as a potential conflict of interest.
